# Antimicrobial use in pig herds in Ireland: analysis of a national database (2019–2023)

**DOI:** 10.1186/s40813-025-00438-5

**Published:** 2025-05-02

**Authors:** Julie Bolton, Lorcan O’Neill, Caroline Garvan, Andrew W. Byrne

**Affiliations:** 1 Antimicrobial Resistance Section, Veterinary Medicines, Antimicrobial Resistance, Animal By Products and Transmissible Spongiform Encephalopathies Division (VMAAT), Department of Agriculture, Food and the Marine (DAFM), Backweston, Kildare, W23 VW2C Ireland; 2 Bacteriology and Parasitology Division, Central Veterinary Research Laboratory (CVRL), DAFM, Backweston Laboratory Campus, Celbridge, W23 VW2C Ireland; 3 Pig Development Department, Teagasc, The Irish Food and Agriculture Authority, Moorepark, Fermoy, Co Cork, Ireland; 4 School of Veterinary Medicine, University College Dublin, Belfield, Dublin 4, Ireland; 5 One-Health Scientific Support Unit, DAFM, Agriculture House, Dublin, D02 WK12 Ireland

**Keywords:** Antimicrobial resistance, Porcine health, One health, Antibiotics, Ireland

## Abstract

**Background:**

Antimicrobial resistance (AMR) in human and animal pathogens remains a global One-Health threat. The associations between antimicrobial use (AMU) and the evolution and dissemination of AMR bacteria, and their resistance genes, highlight the importance of monitoring and regulating AMU. Here, we present an analysis of national monitoring data of AMU in pig facilities in Ireland from 2019 to 2023 via the recently established National AMU Database. AMU was measured using two metrics (mg per corrected population units (mg/PCU) and defined daily dose (DDDvet/PCU)). Temporal trend models were fit using regression models with population average effects given there were multiple observations per herd, while controlling for herd type and size.

**Results:**

Linear spline models revealed no significant change in overall usage from Q1-2019 until mid-2020, followed by a significant decrease in usage until mid-2022. There was evidence of increases in usage from mid-2022 until the end of the time series; the exact timing of the changes in trends varied by the AMU metric. A multinomial logit regression model suggested that there was a significantly decreased probability of premix use relative to oral administration from Q3-2021 through Q4-2023 (OR: 0.70 − 0.58; *P* < 0.03). The predicted probability that a high priority critically important antimicrobial (HPCIA) was used in a herd during a year-quarter declined by an average of 9% per quarter (OR: 0.91; 95% CI: 0.90–0.92; *p* < 0.001) over the study period. The mean decline in use of cephalosporin (3rd /4th generation), fluoroquinolone and macrolide (a former HPCIA) per quarter were estimated to be -12% (95% CI: -8– -15%), -9% (95% CI: -8– -10%) and − 4% (95% CI: -2– -4%), respectively.

**Conclusions:**

This exploration of AMU in pigs in Ireland revealed significant changes in overall usage, with both decreases and increases. There were declines in usage of HPCIA agents. Additionally, there was evidence of a significant decline in the use of oral premixes, coinciding with policy change. Further monitoring of AMU is essential to understand how the pig farming sector is responding to policy changes (e.g., increasing AMU in response to zinc oxide bans).

**Supplementary Information:**

The online version contains supplementary material available at 10.1186/s40813-025-00438-5.

## Background

Antimicrobial resistance (AMR) remains a significant global challenge for human and animal health [1, 2]. Antimicrobial resistance is a one health issue, whereby AMR bacteria and their resistance genes may be preferentially selected through the use of antimicrobials (AMs) in both humans and animals [3]. Global antimicrobial usage (AMU) in livestock has been estimated at tens of thousands of tonnes per annum [4], with future increases forecast [5]. Pig farming is recognised as the sector with the highest AMU in many countries [6–9] and has naturally been a focus of attention for improving antimicrobial stewardship and decreasing use. Several European countries have demonstrated significant reductions of AMU in their respective pig farming sectors in recent years, with national reports claiming reductions of 69% in the UK between 2015 and 2023, 67% in France between 2011 and 2022 and 45% in Belgium between 2018 and 2023 [6–9]. Peer reviewed research has also found positive declining trends in several other countries in Europe (e.g. Switzerland [10], Denmark ([11]), as a consequence of concerted efforts at responsible usage.

Despite this, increased awareness of the dangers of improper usage practices are still required to gain greater improvements. Such warnings focus on the appropriate usage and improving regulation of AMs for food production [12]. For example, the European Union (EU) in 2022 implemented policy changes (e.g. implementation of Regulations EU 2019/6 on veterinary medicinal products [13] and EU 2019/4 on medicated feed [14]). These regulations prohibit the prophylactic use of AMs and, in individual animals only, represent an outright ban on the prophylactic use of AMs in feed and restrictions on certain other applications (e.g. using high priority AMs) (legislation: [13, 14] and see [15] for discussion). Furthermore, legislation sets out that AMs should not be used to overcome other inadequate practices (e.g. poor hygiene, animal husbandry, or management), and the requirement for monitoring of AMU at the farm/species level.

The National Antimicrobial Usage Database (NAMUD) for pigs was officially launched by the Department of Agriculture Food and the Marine Medicine (DAFM) in 2019 [16]. Farmers directly enter their herd AMU data four times per annum on an online portal [17]. Participation is a requirement a national assurance scheme (Bord Bia Farm quality), with the majority of commercial pig farms subscribe (e.g. >95% of herds with 1000 animals or more). The NAMUD facilitates individualised benchmarking reports to herd keepers, as a means of promoting reduced AMU and, where necessary, responsible use.

The analysis of national-level representative datasets is key to monitoring AMU, assessing changes in use and informing responses to national and international policy [18]. Before the establishment of NAMUD, the only published data on AMU in the Irish pig sector were from a cross-sectional study conducted on 67 farrow-to-finish farms [19]. This former study, which was based on data from 2016 and prior to the new EU regulations, estimated that pig farming accounted for 40% of veterinary AMU in Ireland and revealed that AMU was characterised by a high proportion of prophylactic use and was delivered primarily via medicated feed. Here, we present an analysis of data from the initial five years of NAMUD with the objective of analysing national-level AMU data from pigs from a temporal and selected risk factor perspective.

## Methods

### Target population

This study included all pig herds contributing data (by July 2024), for any quarters during the study period, to the NAMUD for pigs between quarter 1, 2019, and quarter 4, 2023. The only eligibility criterion was that information was provided by the herd owner/keeper (the person responsible for entering data for the particular herd) on AMU for a particular year quarter. The initial rollout of the NAMUD was targeted at commercial herds that hold the majority of pigs in Ireland, and was made one prerequisite of the Bord Bia pig quality assurance scheme (Bord Bia, 2021). This is an accredited voluntary scheme (ISO17065) (there is no legal requirement for submission to this database) that sets out the best practice for pig production in Ireland and leads to certification for qualifying herds. Insight into the data collected and entered by participants can be found in DAFM [17]. Herd owners filled in herd/company details, holding identifiers, enterprise types (e.g. finisher/fattening), in-feed and non-feed medication, administration route (e.g. oral), quantities of AMs attributable to each population category (e.g. piglets), breeding animal population, movements off farm by animal types (e.g. sows/gilts, piglets, weaners/growers), and the number of pigs going to slaughter.

Herd types were categorised according to the type of enterprise inputted by the participating farmers as follows: farrow-to-finish finishing/fattening units, breeder-to-weaner units or “other”. Typically, breeder-to-weaner farms in Ireland move pigs off the farm when they are ready for transfer to a finishing/fattening unit, while finishing/fattening units usually receive pigs from a single source.

### Database metrics

The milligram per kilogram population corrected unit (mg/PCU) is the indicator of antimicrobial consumption used by the NAMUD for pigs (note, for the benefit of communications with stakeholders, this metric is described as “mg/kg” within the database). This metric is analogous to the mg/PCU indicator used by the European Medicines Agency (EMA) to report sales of AMs in the EU/EEA which uses the weight of the active ingredient (mg) as the numerator and the weight of pigs (kg) as the denominator. The denominator used by NAMUD is calculated at farm level using the assigned weights for each category of pig as defined by the EMA: 240 kg per breeding sow, 65 kg per finisher pig sold from the farm (i.e., sold to another farm or sent to slaughter) and, 25 kg per weaner pig sold from the farm [20].

We also calculated a second metric based on Daily Defined Dose (DDD). This analysis used the Daily Defined Dose per population correction unit (DDDvet/PCU), which was calculated following European Medicines Agency guidelines [20, 21] and can be found in the supplementary material. The DDDvet numerator uses doses assigned by the EMA to each AM and route of administration and thus accounts for differences in potency between different AMs [22].

### Data management

Electronic data were obtained for pig herds from the NAMUD, with data on usage provided on a herd-quarter basis for each antimicrobial and each administration route (oral, premix and parenteral) from quarter 1, 2019, to quarter 4, 2023. An initial selection screened out all herds that had no AMU data for any given quarter during the study period. All the data were received in CSV format before being merged and restructured via Stata 16 MP [23]. Depending on the analysis type, the data were collapsed to herd, herd-quarter, or herd-quarter-administration levels (the “experimental unit”).

### Statistical analyses

#### Trend analysis

To establish whether there were temporal changes in the raw mean herd-level AMU in mg/PCU changes over quarters, trend models were fitted. Given that there were repeated measures from herds, the models were fitted via a population-averaged (PA) framework (generalised estimating equation (GEE) model [24]) to control for the nonindependence of observations clustered within herds via an exchangeable correlation matrix structure [25, 26]. This model estimates the average response across the population, which was our interest (in comparison with subject-specific models [25]). We used a gamma distribution and log link to model usage on continuous scales, given the non-normal distributions of the outcome variables. Competing models were compared via the quasilikelihood information criterion (QIC), an indicator similar to Akaike’s information criterion (AIC) that can be applied to nonlikelihood-based PA models [24]. The models with the lowest QIC values were considered those with the greatest support [24]. Year quarters were fitted as categorical dummy variables (predictors). Interquarter comparisons were tested within the model via Wald tests. Second, a linear-spline model was fitted to the data to model the trend in the data (also known as a “broken stick” model), assuming one or two breakpoints (knots) between quarters across the time series, as used elsewhere to model temporal trends (e.g [27]). Breakpoints were chosen on the basis of the inflection point from the categorical model, but different breakpoints were compared (using QIC) by assessing the fit of the competing spline models.

Given that the population varied over time, as not all herds were represented across all the time series, the trend analysis controlled for the potential confounders. Both herd size and type were forced into the model irrespective of whether being significant. The multivariable model was developed to control for these potential effects, with year–quarter fit as a categorical variable, linear predictor and as a linear spline, respectively, to assess adjusted mean AMU over time.

Models were fitted with (A) all data and (B) restricted to herds that reported AMU for each quarter of the time series. Predictions with estimated 95% CIs were computed via the MARGINS command in Stata 16 MP [23].

#### Changes in AM route of administration over time

Changes in how AMs were administered over time at the herd-quarter level were assessed via a multinomial logit regression model [28, 29]. In pig production, antimicrobials are primarily administered via oral or parenteral routes. Oral medications may be administered using water or feed. However, there is a distinction between the use of oral premix in medicated feed (which must be manufactured by a licensed feed mill) and the use of oral remedies in feed or water which may be administered by the farmer (Regulation (EU) 2019/4). Therefore, the three administration routes assessed were, oral premix, oral and parenteral with the *a priori* hypothesis that the probability of oral premix administration occurring during a quarter would decline over time relative to oral or parenteral routes. This approach models the relative proportion of administrations of AM with a 3-level variable representing the reporting of any oral, parenteral and oral premix routes via a dichotomised outcome at each herd quarter within the dataset. For example, a herd would be coded “1” during a given quarter for premix administration where any premix AMU was reported or “0” if no premix AMU was reported. Therefore, the relative proportions reported are not equivalent to the proportions of administrations within herds that could be attributable to each route, as *animal-administration level* data were not available within the NAMUD.

Since herds were represented within the time series more than once, cluster-adjusted robust standard errors were employed via the CLUSTER option within the MLOGIT suite. This model outcome was compared with a multinomial random effects model, as implemented with the generalised structural equation modelling (GSEM) suite in Stata 16 [30]. To aid interpretation, parameters were reported as relative risk ratios, which were derived by exponentiating the logit coefficients.

#### Changes in the use of highest priority critically important antimicrobials

The change in the use of highest priority critically important antimicrobials (HPCIA [31]) over time was modelled as a binary outcome via a population averaged mixed effect logit model, with time (quarters) modelled as both categorical and linear predictors. Herd was fitted as a random effect, with fixed effects for herd type and size. In the dataset, the HPCIA classes used on the study farms included, fluoroquinolones (enrofloxacin and marbofloxacin), 3rd /4th gen cephalosporins (ceftiofur) and polymyxins (colistin). Individual models for each AM class were also fitted to explore which classes may drive any changes in HPCIA use. In addition, models of macrolides (tildipirosin, tilmicosin, tulathromycin, tylosin, and tylvalosin), which had previously been classed as a HCPIA, were explored. The European Medicines Agency (EMA) classifies fluoroquinolones, polymyxins and 3rd- and 4th-generation cephalosporins as category B antimicrobials (‘restrict’) and macrolides as category C (‘caution’). In 2024, the WHO published a List of Medically Important Antimicrobials intended as a risk management tool for mitigating antimicrobial resistance due to non-human use which saw macrolides being reclassified from HPCIA to CIA by the WHO after a thorough review [32]. However, for the purposes of the present study, we fitted models to the macrolide data.

## Results

### Descriptive results

Overall, the dataset contained 23,996 observations, with each observation relating to the use of an AM/herd/quarter for each administration route (oral, premix, or parenteral). There were 4,498 year–quarter observations from 359 herds, meaning that the average herd had 12.5 quarterly reports within the dataset (median: 13; IQR: 8–17; max: 20). Fifty-seven herds (16%) submitted data for all quarters over the five years, whereas 146 (41%) submitted a minimum of 16 quarters (four years). The median herd began reporting during year 2 (2020; IQR: 1–3), with 71% (*n* = 255) of herds having reported by the end of that year.

A total of 278 herds with > 1000 animals were represented within the dataset, from a total population estimated from data from a national pig census of 281 (98.9%) [33]. Taking the average breeding herd size reported per quarter for each herd and summing across all herds, an average of 148,248 breeding pigs were reported. In 2019, only a mean of 64,989 breeding animals were reported, representing 45% of the national breeding herd; however, from 2020 to 2023, the coverage exceeded 78% of the census figure.

The population (based on the average number of breeding animals present per quarter and the total number of animals sent to slaughter) per quarter varied from 373,776 to 482,953 in 2019 when the database was being established, increasing dramatically thereafter, with a range of 766,942–1,014,414 (Supplementary material Fig. S1). In addition, the average total number of animals moved per quarter varied by animal type: the mean number of breeding animals moved per quarter was 6,111 (quarterly range: 1,703–13,784), the number of piglets moved per quarter was 63,154 (quarterly range: 39,080–98,137), and the average number of weaner/growers moved per quarter was 190,153 (quarterly range: 87,701 − 300,793). The maximum number of herds contributing data during a quarter was 293 (82% of all farms that contributed data over the time series; Q1–2022).

Among the herds within the study population, 50% (*n* = 178) were reported as farrow-to-finish units, 39% (*n* = 140) were finishing/fattening units, 9% (*n* = 33) were breeder-to-weaner units, and the remaining 8 herds were classified as “other” production units (Table 1). Information on the variation in herd size and composition across herd types is presented in Table 1.

Summary statistics for overall AMU is presented in Table 1, and for of each AM compound/class are presented in the supplementary material for mg/PCU (Table S1) and DDDvetPCU (Table S2). Visual trends for each AM per quarter are presented in Figures S2-S5.

**Table 1:** Summary statistics on pig herd types and herd sizes across year quarters. AMU: antimicrobial usage

Herd type	Farrow-to-finish unit	Breeder to Weaner unit	Finisher/fattening unit	Other	Total
Herds-n	178	33	140	8	359
Observations	2,639	435	1,334	90	4,498
Herd size					
Mean	4359	1101	2468	2449	3445
Median	3158	829	1697	1407	2300
25th%ile	1445	562	766	429	972
75th%ile	5820	1425	3177	4500	4650
Breeding pigs					
Mean	630	886	15	298	466
Median	499	730	0	191	290
25th%ile	220	430	0	0	0
75th%ile	819	1200	0	450	680
AMU (mg/PCU)					
Mean	80.9	116.8	34.5	49.0	70.0
Median	25.9	36.5	1.1	22.9	12.3
25th%ile	4.4	4.8	0.3	1.1	1.3
75th%ile	94.0	151.6	12.9	61.4	78.6
AMU (DDDvetPCU)					
Mean	5.4	21.9	2.2	2.6	6.0
Median	1.9	7.3	0.1	1.0	1.1
25th%ile	0.4	1.2	0.0	0.1	0.1
75th%ile	6.0	33.1	0.9	4.6	5.5

### Adjusted Temporal trend– mg/PCU

The final categorical and spline models explained significant variation in the outcome (joint Prob > χ^2^ < 0.001). Overall, herd size did not explain significant variation in the outcome (post hoc Wald test: χ (DF: 3) > 6; Prob > χ^2^ > 0.09) but was retained within both final models to control for potential confounding effects. Herd production type significantly explained the variation in AMU (χ^2^(DF: 2) = 28.94; Prob > χ^2^ < 0.001), with significantly lower AMU in fattening/finishing units than in farrow-to-finish units (categorical model: RR: 0.49; *p* < 0.001; spline: RR: 0.49; *p* < 0.001) or breeder-to-weaner units (categorical model: RR: 0.31; *p* < 0.001; spline model: β: 0.32; *p* = 0.001).

**Fig. 1:**
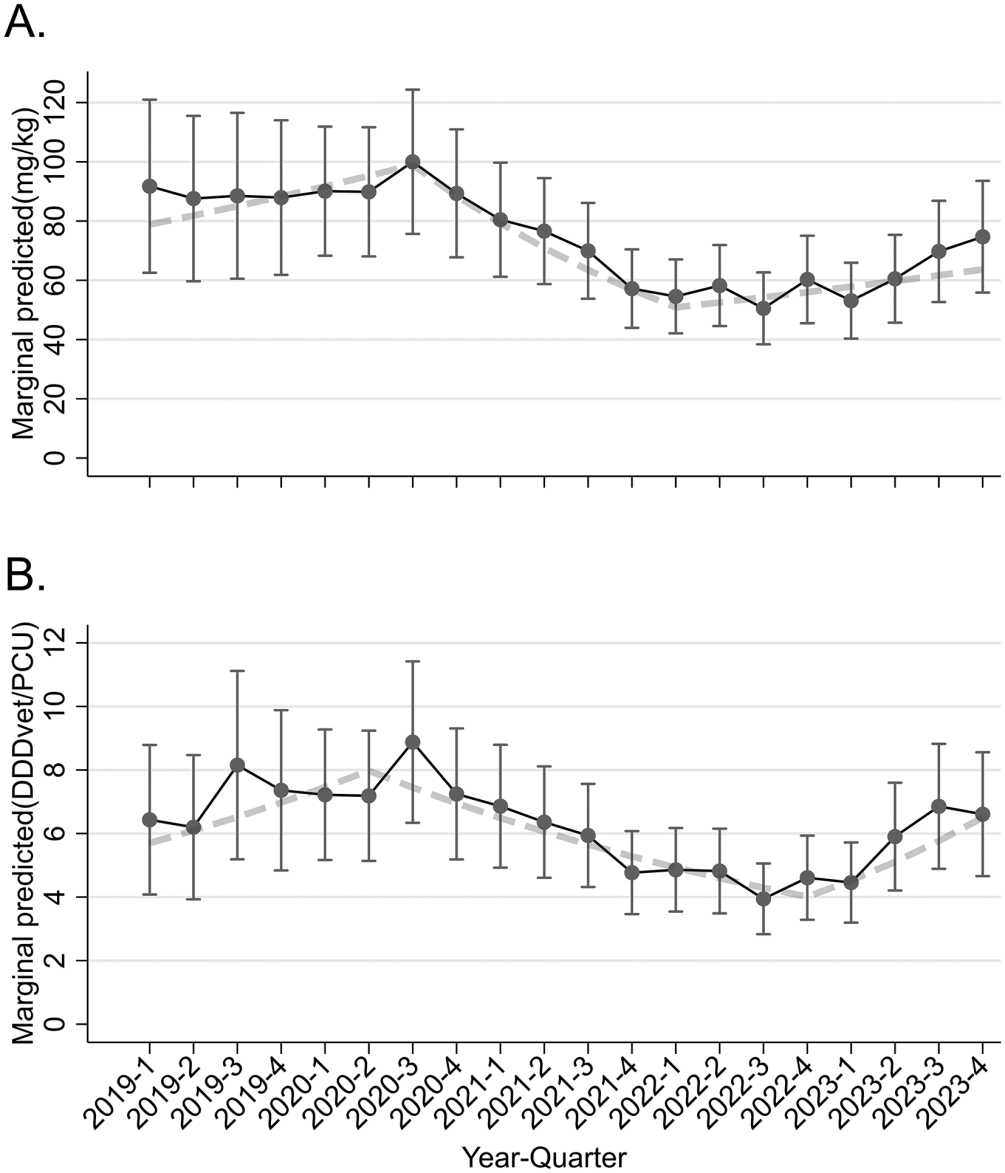
Relationship between quarter and the marginal predicted AMU measured as (**A**) mg/PCU and (**B**) DDDvet/PCU from a multivariable model controlling for herd type and size. Dashed line: predictions from a linear spline model; points and error bars: predictions from a multivariable categorical GEE regression model with associated 95% CIs

Modelling time as a categorical variable (per quarter) suggested that there was a period of nonsignificant change in AMU (Q1-2019 to Q3-2020; mean predicted AMU range: 87.6 mg/PCU to 100.0 mg/PCU) before a significant decline (from 100.0 mg/PCU to 50.5 mg/PCU) before increasing again to 74.7 mg/PCU in Q4-2023 (Fig. 1). Controlling for covariables, the lowest mean marginal predicted usage was found in Q2-2022 at 50.5 mg/PCU (95% CI: 38.4–62.7 mg/PCU), and the highest usage was reported in Q3-2020 at 100.0 mg/PCU (95% CI: 75.7–124.4 mg/PCU). Usage was significantly lower in each quarter from Q2–2021 to Q4–2023 relative to the peak usage (*P* < 0.04; referent Q3–2020; Table 2).

The most supported spline model (based on the QIC) had cut-off points at Q3–2020 and Q1–2022. The first linear spline from Q1–2019 to Q3–2020 had a nonsignificant positive coefficient (RR: 1.02; *p* = 0.253), followed by a significant decline in AMU from Q4–2020 to Q1–2022 (*p* < 0.001). For each subsequent quarter, the mean mg/PCU decreased by 10% (95% CI: 7.1–12.0% decline per quarter). Finally, there was an increasing trend thereafter, with a 4% increase in mean usage per quarter (RR: 1.04; 95% CI: 1.01–1.06).

A similar pattern was found when the dataset was restricted to only herds that were represented in all quarters (*n* = 1140; herds = 57; Fig. S6). The final multivariable categorical model suggested that AMU was significantly lower in Q1–2021 (RR: 0.61; *p* = 0.021) and from Q2–2021 to Q4–2023 (RR range: 0.59–0.45); *p* < 0.020) than in Q2–2020. The predicted mean marginal AMU from this model peaked at Q3–2020 at 83.5 mg/PCU (95% CI: 51.1–116.0) and declined to 37.6 mg/PCU (95% CI: 23.0–52.2) at its nadir in Q1–2022.

Fitting a spline model suggested that the first linear spline exhibited a significant increasing trend in usage (RR: 1.06 per quarter; *p* = 0.012), followed by a significant decline from Q4–2020 to Q1–2022 (RR: 0.89 per quarter; *p* < 0.001), before a nonsignificant trend until Q4–2023 (RR: 1.04; *p* = 0.054; Fig. S7).

**Table 2:** Generalised estimating equation model of the AMU Mg/PCU and DDDvet/PCU variation in pig herds in Ireland across the year quarters over the study period (2019–2023); distribution = gamma; link function = log

	mg/PCU		DDDvet/PCU	
	EXP(β)^1^ (95% CI)	*p* value	EXP(β)^1^ (95% CI)	*p* value
Year-quarter				
2019-1	0.92 (0.66–1.28)	0.616	0.72 (0.5–1.05)	0.091
2019-2	0.88 (0.63–1.23)	0.440	0.7 (0.48–1.01)	0.060
2019-3	0.89 (0.63–1.24)	0.475	0.92 (0.63–1.33)	0.654
2019-4	0.88 (0.64–1.21)	0.424	0.83 (0.58–1.18)	0.294
2020-1	0.9 (0.69–1.17)	0.440	0.81 (0.61–1.09)	0.168
2020-2	0.9 (0.69–1.17)	0.429	0.81 (0.6–1.09)	0.160
2020-3 (Referent)				
2020-4	0.89 (0.69–1.16)	0.403	0.82 (0.61–1.1)	0.176
2021-1	0.8 (0.62–1.05)	0.105	0.77 (0.58–1.03)	0.083
2021-2	0.77 (0.59–0.99)	0.043	0.72 (0.54–0.95)	0.023
2021-3	0.7 (0.54–0.9)	0.006	0.67 (0.5–0.89)	0.006
2021-4	0.57 (0.44–0.74)	< 0.001	0.54 (0.4–0.71)	< 0.001
2022-1	0.55 (0.42–0.7)	< 0.001	0.55 (0.41–0.73)	< 0.001
2022-2	0.58 (0.45–0.76)	< 0.001	0.54 (0.41–0.72)	< 0.001
2022-3	0.51 (0.39–0.66)	< 0.001	0.44 (0.33–0.6)	< 0.001
2022-4	0.6 (0.46–0.79)	< 0.001	0.52 (0.38–0.7)	< 0.001
2023-1	0.53 (0.41–0.69)	< 0.001	0.5 (0.37–0.67)	< 0.001
2023-2	0.61 (0.46–0.79)	< 0.001	0.66 (0.49–0.9)	0.008
2023-3	0.7 (0.53–0.91)	0.009	0.77 (0.57–1.04)	0.091
2023-4	0.75 (0.57 - NA)	0.039	0.74 (0.55–1.01)	0.060
Herd type				
Breeder to Weaner unit (Ref.)				
Farrow-to-finish	0.64 (0.38–1.09)	0.099	0.25 (0.15–0.43)	< 0.001
Finisher/fattening unit	0.31 (0.18–0.53)	< 0.001	0.12 (0.07–0.2)	< 0.001
Other	0.44 (0.15–1.3)	0.136	0.16 (0.05–0.5)	0.001
Herd size quartiles (range)				
1 (< 1442; Ref.)				
2 (1442–3157)	1.07 (0.86–1.34)	0.529	1.04 (0.82–1.32)	0.744
3 (3158–6006)	1.06 (0.82–1.38)	0.650	0.84 (0.64–1.11)	0.231
4 (6001–23056)	1.08 (0.79–1.48)	0.611	0.91 (0.65–1.27)	0.572
Constant	166.12 (100.07–275.78)	< 0.001	32.28 (19.2–54.27)	< 0.001

^1^ exponentiated coefficients are relative ratios (RR)

### Adjusted Temporal trend– DDDvet/PCU

The final multivariable model where quarters were fitted as categories is presented in Table 2, with broadly similar trends to those of the mg/PCU model (Fig. 1). There was significantly lower mean usage relative to Q3–2020 from Q2–2022 through Q2–2023 (*p* < 0.03). However, there was no significant difference between Q3 and Q4 2023 relative to Q3–2022 (p > = 0.06). A linear spline model with two knots suggested a nonsignificant increase between Q1-2019 and Q2-2020 (RR: 1.04 per quarter; *P* = 0.09), followed by a significant decrease of 7% per quarter until Q3-2022 (RR: 0.93 per quarter; *P* < 0.001), and finally a significant increasing trend of 15% per quarter until Q4-2023 (RR: 1.15 per quarter; *P* < 0.001).

### Compositional changes in AMs over time

**Fig. 2:**
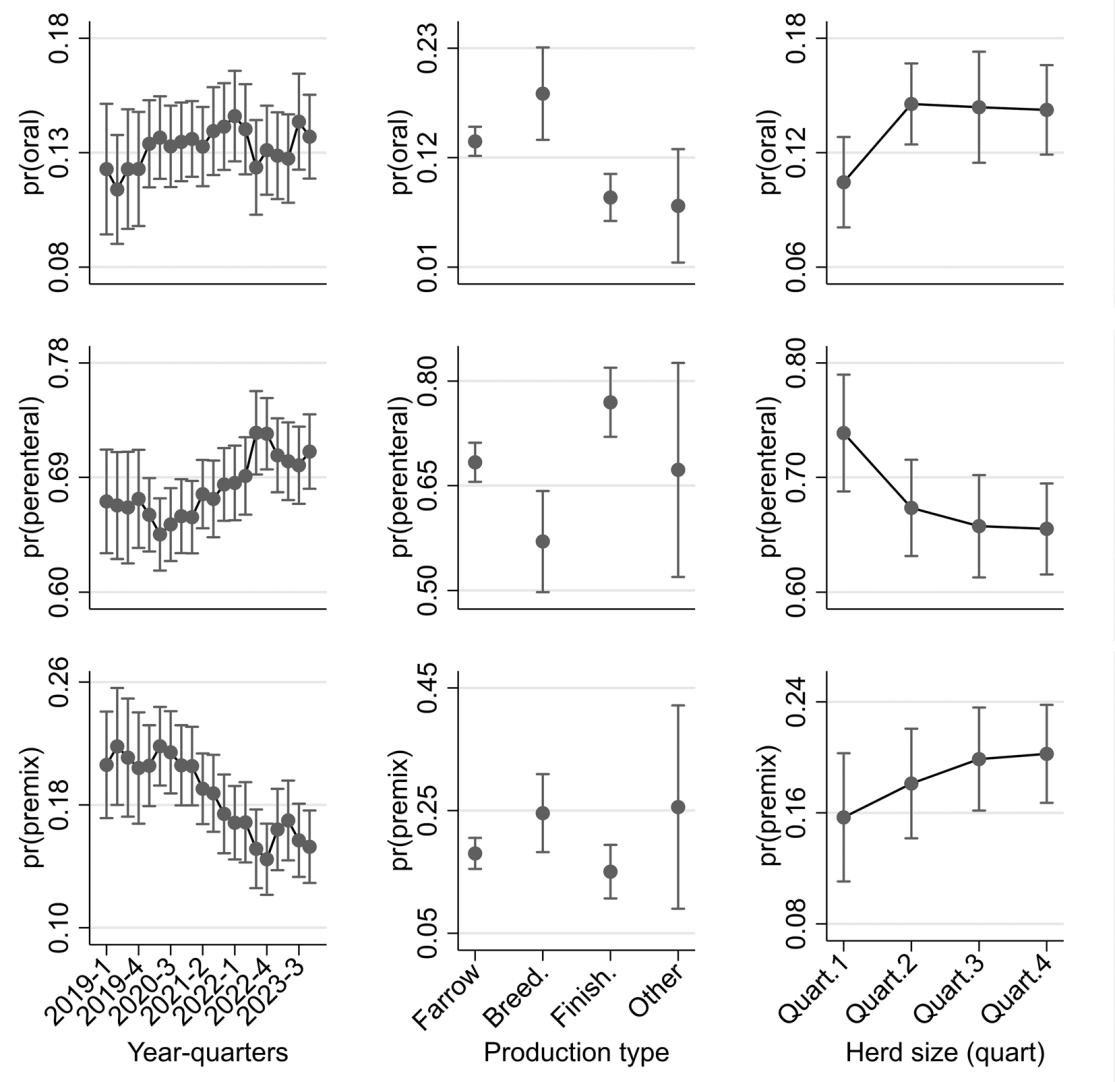
Predicted marginal probabilities from a multinomial logit model of the administration (oral, parenteral, or oral premix) of AMs across year quarters for different pig herd types (farrow-to-finish (farrow), breeder-to-weaner (breed), finisher/fattening unit (finish) and other, and herd size (quartiles)

Using AMU data organised by route of administration level, the proportions of administrations among the three routes (categorised as oral, parenteral and oral premixes) across quarters were analysed via a multivariable multinominal logit regression model. The model predictions (Fig. 2) revealed that there was a decrease in the relative proportion of premix administrations over time, especially in 2022, relative to previous years. The relative log odds of oral premix administration significantly declined in comparison with those of oral administration at the base (referent) period (Q2 2019) and Q3 2021 (relative risk ratio (RRR): 0.70; *p* = 0.028) through Q4 2023 (RRR: 0.58; *p* < 0.001; Table S3), with all other variables remaining constant in the model. There was also a trend towards a reduced relative risk of oral premix use relative to parenteral administration between baseline (Q2 2019) and Q2 2022–Q4 2023 (RRR < 0.78; *p* < 0.04).

There was significant variation in the proportion of administrative routes used across production types (Table S3). Breeder-to-weaner herds had the highest probability of oral administration, whereas parenteral administration had a higher probability of use in finisher herds (Fig. 2). There was less evidence of large variation in oral premix use among herd types. The probability of parenteral, relative to oral, use in breeder-to-weaner herds was significantly lower than that for farrow to finish units (RRR: 0.62; *p* = 0.010) but significantly greater for finisher herds (RRR: 1.93; *P* < 0.001; Table S3). The proportion of administrations that were premixes, relative to parenteral, increased with increasing herd-size (Fig. 2), though not significantly so ((RRR: 1.27–1.46; *P* = 0.108–0.278). Compared with parenteral administrations, larger herds tended to have higher relative probabilities of oral use than herds in the first quartile (< 1442 total animals) of herd size (RRR: 1.53–1.55; *P* < 0.03; Fig. 2).

Similar results were found when the data were fitted within a GSEM framework (Supplementary material: Fig. S8; Table S4).

### Changes in the use of HPCIAs over time

**Fig. 3:**
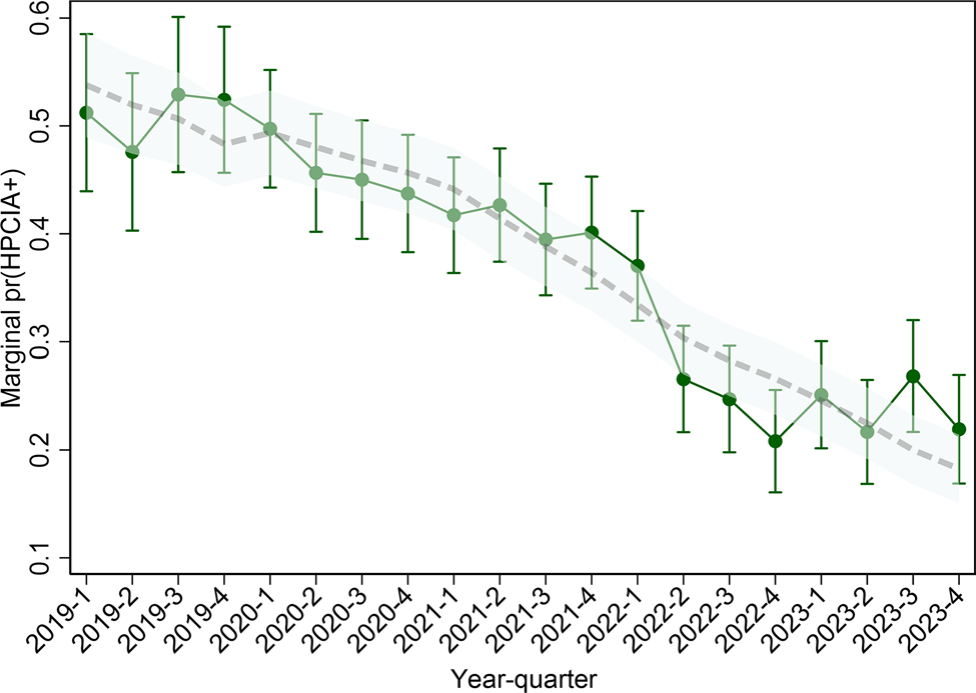
Predicted probability that an HPCIA is used per quarter from a logistic population-averaged mixed effect model. The solid connected line represents the predictions from a model where time is fitted as a categorical variable, with associated 95% CI spikes. The dashed line is the marginal prediction from the linear spline model with two knots, adjusted for production type and herd size; the transparent green range is the 95% CI for the spline prediction

Over the study period, there was a significant decline in the use of HPCIAs. The odds of HPCIA use declined by an average of 9% per quarter when quarter was treated as a linear predictor across the time series (OR: 0.91; 95% CI: 0.90–0.92; *p* < 0.001; Fig. 3). A model treating the quarter-year as a categorical variable predicted a marginal probability high of 52.9% (95% CI: 45.7-60.1%) for Q3-2019, which decreased to a low of 21.7% (95% CI: 16.9-26.5%) in Q2-2023. A spline model with a knot at Q4-2022 suggested that the trend was composed of a decline of 9.6% per quarter (*p* < 0.001) from 2019 to 2022, and then declining trend of 5.9% per quarter until the end of 2023 (*p* = 0.04; Fig. 3).

**Fig. 4:**
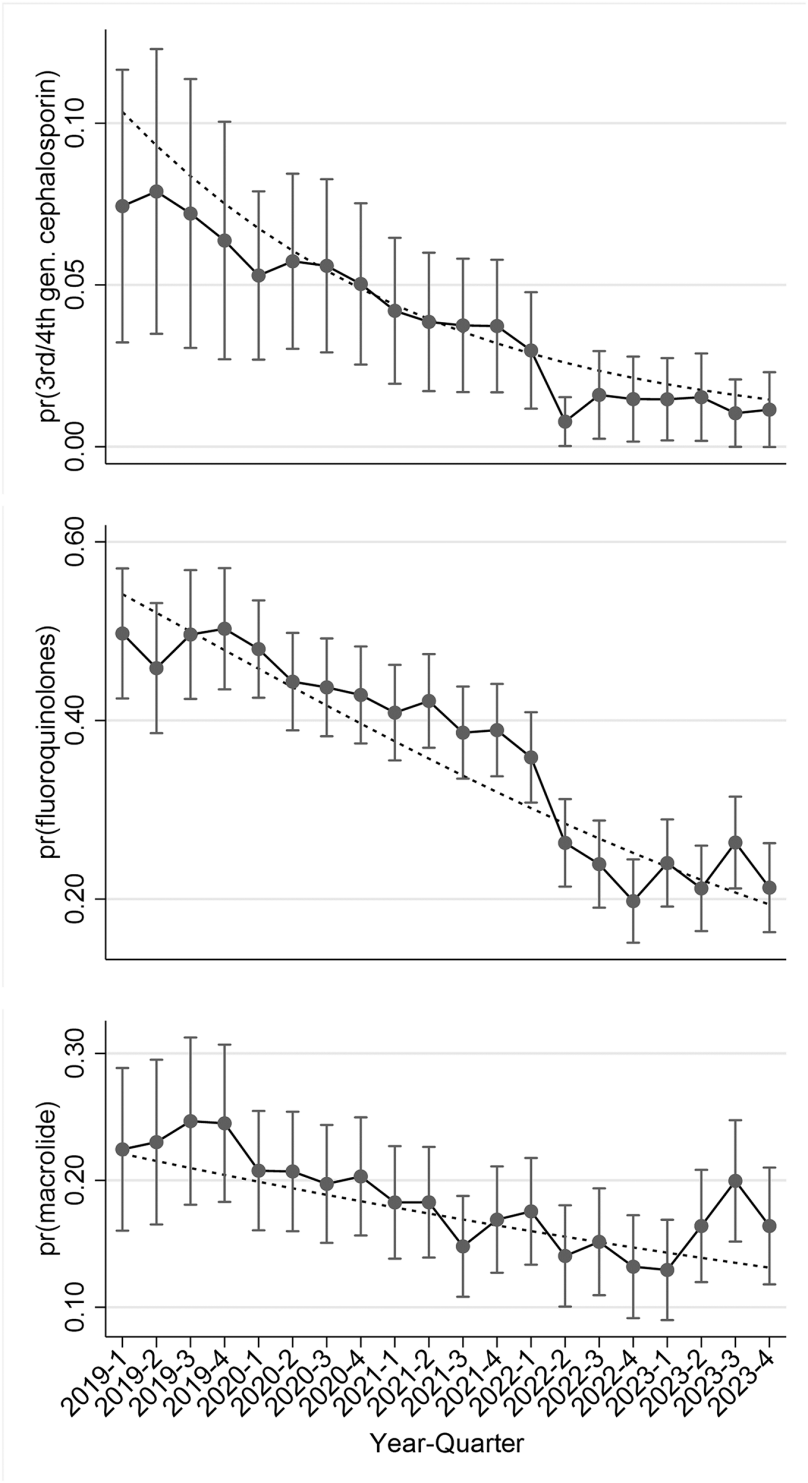
Predicted probabilities from separate logistic regression models for the use per quarter of HPCIAs − 3rd /4th generation cephalosporins, fluoroquinolones, and macrolides

Negative trends over time in the probabilities of HPCIA use per quarter for each of the 3rd /4th generation cephalosporin, fluoroquinolone families, and also macrolides were found (Fig. 4). The 3rd /4th generation cephalosporin usage declined significantly, by an average of 12% per quarter (OR: 0.88 per quarter; 95% CI: 0.85–0.92; *p* < 0.001). The categorical model suggested a significant decline in the probability of use from Q3–2021 onwards relative to the peak usage probability from Q2–2021 (*p* < 0.05). Multivariable models for fluoroquinolones (OR: 0.91 per quarter; 95% CI: 0.90–0.92; *p* < 0.001) and macrolides (OR: 0.96 per quarter; 95% CI: 0.96–0.98; *p* < 0.001) showed significant negative linear trends in usage odds of 9% per quarter and 4% per quarter, respectively. For fluoroquinolones, a significantly lower probability of use occurred from Q4–2020 to Q4–2023 compared to Q4–2019 (OR: 0.74–0.20; *P* < 0.05). For macrolides, a significantly lower probability of use relative to the peak (Q3-2019) occurred from Q3–2021 to Q4–2023 (*p* < 0.02), with the exception of Q3–2023, which was not distinguishable from Q3–2019 (OR: 0.75; 95% CI: 0.50–1.14; *p* = 0.177) (Table 3).

**Table 3:** Models fitting the probability of use per herd quarter of a HPCIA 3rd /4th generation cephalosporins. Notably, cephalosporins were not used in “other” production types and were therefore omitted from the final model

	a. Pr(macrolide use/quarter)		b. Pr(fluoroquinolone use/quarter)		c. Pr(cephalosporin use/quarter)	
	EXP(β) (95% CI)	*p* value	EXP(β) (95% CI)	*p* value	EXP(β) (95% CI)	*p* value
Year-quarter						
2019-1	0.878 (0.551-1.400)	0.585	0.975 (0.650–1.464)	0.904	0.932 (0.389–2.234)	0.874
2019-2	0.909 (0.57–1.447)	0.686	0.813 (0.542–1.219)	0.316	ref (-)	
2019-3	ref (-)		0.97 (0.648–1.453)	0.882	0.898 (0.372–2.168)	0.810
2019-4	0.990 (0.636–1.543)	0.966	ref (-)		0.776 (0.323–1.865)	0.571
2020-1	0.791 (0.528–1.186)	0.257	0.899 (0.637–1.267)	0.542	0.624 (0.279–1.395)	0.251
2020-2	0.788 (0.526–1.182)	0.250	0.757 (0.537–1.068)	0.113	0.685 (0.31–1.513)	0.350
2020-3	0.740 (0.491–1.113)	0.148	0.735 (0.520–1.038)	0.080	0.665 (0.3-1.477)	0.316
2020-4	0.769 (0.512–1.153)	0.203	0.706 (0.500-0.996)	0.047	0.588 (0.262–1.319)	0.198
2021-1	0.670 (0.445–1.009)	0.055	0.643 (0.456–0.905)	0.011	0.476 (0.206–1.099)	0.082
2021-2	0.671 (0.447–1.006)	0.054	0.684 (0.487–0.960)	0.028	0.43 (0.184–1.009)	0.052
2021-3	0.516 (0.340–0.782)	0.002	0.577 (0.411–0.810)	0.002	0.416 (0.178–0.972)	0.043
2021-4	0.608 (0.404–0.915)	0.017	0.585 (0.417–0.822)	0.002	0.414 (0.177–0.966)	0.041
2022-1	0.637 (0.425–0.955)	0.029	0.504 (0.359–0.708)	< 0.001	0.316 (0.129–0.776)	0.012
2022-2	0.484 (0.316–0.741)	0.001	0.305 (0.214–0.435)	< 0.001	0.046 (0.007–0.319)	0.002
2022-3	0.531 (0.347–0.814)	0.004	0.266 (0.185–0.383)	< 0.001	0.145 (0.043–0.491)	0.002
2022-4	0.450 (0.289-0.700)	< 0.001	0.205 (0.140–0.298)	< 0.001	0.13 (0.035–0.475)	0.002
2024-1	0.439 (0.283–0.682)	< 0.001	0.268 (0.186–0.385)	< 0.001	0.129 (0.036–0.459)	0.002
2024-2	0.585 (0.382–0.896)	0.014	0.225 (0.155–0.327)	< 0.001	0.137 (0.038–0.49)	0.002
2024-3	0.751 (0.496–1.138)	0.177	0.306 (0.213–0.441)	< 0.001	0.077 (0.015–0.397)	0.002
2024-4	0.586 (0.379–0.906)	0.016	0.226 (0.154–0.331)	< 0.001	0.09 (0.019–0.435)	0.003
Herd type						
Farrow-to-finish (Ref.)	ref (-)		ref (-)		ref (-)	
Breeder to Weaner unit	1.059 (0.532–2.109)	0.871	1.669 (0.956–2.913)	0.072	1.791 (0.578–5.551)	0.313
Finisher/fattening unit	0.364 (0.218–0.606)	< 0.001	0.205 (0.136–0.309)	< 0.001	0.195 (0.059–0.647)	0.008
Other	0.549 (0.126–2.395)	0.425	0.525 (0.169–1.636)	0.267	no obs. (-)	
Herd size quartiles (size range)						
1 (< 1442; Ref.)	ref (-)		ref (-)		ref (-)	
2 (1442–3157)	1.314 (0.944–1.828)	0.105	1.506 (1.153–1.967)	0.003	1.009 (0.407–2.498)	0.985
3 (3158–6006)	2.033 (1.421–2.909)	< 0.001	1.939 (1.434–2.621)	< 0.001	3.658 (1.595–8.387)	0.002
4 (6001–23056)	1.946 (1.283–2.951)	0.002	2.317 (1.628–3.298)	< 0.001	4.025 (1.676–9.667)	0.002
Constant	0.293 (0.181–0.472)	< 0.001	1.026 (0.688–1.531)	0.898	0.049 (0.019–0.132)	< 0.001

## Discussion

The present study is the first analysis of AMU data from the NAMUD for pigs and demonstrates that there has been significant variation in AMU over time in pig herds in Ireland and, most importantly, that there has been a quantifiable decline in AMU in response to policy change and programs aimed at reducing AMU usage as part of a broader One Health initiative. The paper also shows how there has been a change in the administration routes of AMs over time, again in response to policy changes. The data presented clearly provide evidence for action for stakeholders to continue reducing AMU. Despite the overall positive picture, there was evidence to suggest an increasing trends in overall usage in the final year of the time-series, coinciding with the ban on the use of high-dose zinc oxide in pig diets within the EU.

The models demonstrated a significant decline in AMU, with an inflection point occurring approximately in quarter 3, 2020, and significant reductions for each subsequent quarter until mid-2022. This reduction coincided with policies brought forward from Ireland’s National Action Plan on AMR (iNAP; gov.ie - Antimicrobial Resistance (AMR) (www.gov.ie)). These improvements mirror progress made elsewhere in the European pig sector [34, 35] and trends reported elsewhere at the national level, such as in the UK [8]. Furthermore, there was a significant decline in the use of oral premixes relative to oral administration in Q1 and Q2 of 2022. The overall reduction in AMU and reduction in oral premix use are linked. Prior to the implementation of the EU regulations in 2022, routine prophylactic use of medicated feed accounted for the majority of AMU within the sector [13, 19]. Since the implementation of these regulations, prophylactic AMU is no longer permitted, and metaphylactic AMU is no longer allowed without sufficient justification. While these restrictions do not prevent AMU *per se*, they present an increased administrative burden, and as a result, veterinarians and farmers have had to adapt their respective prescribing and AMU practices, especially with regard to the use of oral premixes in medicated feed. Indeed, differences in regulatory, administrative and practical workloads were suggested as an explanation for the observation that farms that mill their own feed (and thus have relatively restricted access to medicated feed) had lower AMU than farms that sourced their feed from commercial mills [36]. However, the fact that AMU began to decrease 18 months before the implementation of the new EU regulations suggests that farmers and veterinarians began preparing for the new regulatory environment well in advance, or may reflect a growing awareness of the importance of AMR and better antimicrobial stewardship within the sector. On the other hand, the declining trend in AMU reversed from mid-2022 onwards, coinciding with the EU-wide ban on therapeutic use of ZnO in pig diets. Previously zinc oxide was widely used on Irish pig farms [35], as it was in other pig producing countries [36], to control post weaning diarrhoea. The increase in AMU observed after its prohibition may be due to the use of antimicrobials as an alternative control strategy and other countries have reported increased AMU (e.g., Denmark, and UK) in their respective pig sectors since 2022. However, previous research found no association between zinc oxide and AMU [37]. Further research would be required to verify whether the increased population-level usage was related to the ZnO ban, and it should be noted that AMU levels in 2023 were still below the peak observed in Q3 of 2020.

The analysis presented here also revealed a significant reduction in the use of HPCIAs. In contrast with the overall AMU pattern, there was a more gradual and sustained linear reduction from at least Q3 2019. Current policy guidelines in Ireland state that HPCIAs, which include fluoroquinolones, 3^rd−^/4th -generation cephalosporins, macrolides and colistin, should not be used as first-line treatments in animals [38] and should be used only in exceptional cases after culture and susceptibility testing are completed. Increasing awareness of the importance of better antimicrobial stewardship among farmers and adherence to these guidelines may explain the decreasing trends observed in this study.

Herd size was not associated with AMU in this study. While other studies have reported positive associations between herd size and AMU [39, 40], the findings here are in agreement with a previous study on Irish pig farms [34] and elsewhere [37]. Herd type was associated with AMU in this study. There was lower AMU associated with finishing herds compared to farrow-to-finish herds or breeder-to-weaner farms. Higher AMU levels were observed on breeder-to-weaner units than on farrow-to-finish units, although not significantly different using the mg/PCU metric (*p* = 0.099). This may be explained, in part, by the difference in denominators: since the former category moves pigs off the farm at an earlier age, the denominator for pigs moving off the farm is lower (25 kg vs. 65 kg) and hence the AMU is higher. Higher AMU on farrow-to-finish and breeder-to-weaner farms than on finisher farms was an expected finding in this study and was also reported in a Swiss study (Echtermann et al., 2019). This can be explained by the greater use of AMs during the weaner stages. While accurate attribution to the stage of production is not possible with these data, a previous study showed that 69.7% of all AMs used on Irish pig farms were administered to weaner pigs (O’Neill et al., 2020a), which is consistent with findings in Europe and worldwide (use DANMAP, SDa, Sjollund, and [41, 42]. On the other hand, finisher farms receive pigs after the weaning stage and thus are less likely to receive antimicrobial agents. Notably, in the Irish pig sector, finisher units are usually linked to a single breeder unit and thus do not mix pigs from different sources, a practice that has been associated with higher AMU in finisher pigs in other studies [35, 39, 43].

### Limitations

The present study relied on self-reported data from herd owners/keepers, which may be prone to error and potentially to reporting, or other unknown, bias.Another limitation was the changing population encompassed within the dataset over time. Early in the study, corresponding with the establishment phase of the quality scheme, the population was smaller and may not have fully represented the totality of the target population (commercial pig herds), although with increasing participation, the dataset would have become more representative over time. However, there was a slight decline in the number of herds submitting data towards the end of the time series. While we do not know why some herds were missing data submissions, we speculate that this could relate to lags in data capture and submission, reviews and updates of data, and because of any reviews arising from scheduled and unscheduled audits undertaken as part of the quality scheme. The additional model focusing on herds that participated throughout the study also allowed us to assess the trends for this cohort with a full time series.

This study used two indicators of AMU for the purpose of analysis. The use of different indicators can hamper comparisons between countries (or studies) which use their own defined AMU indicators (e.g., Denmark [6], or the Netherlands [9]. The mg/PCU (which is the actual metric used by NAMUD) and the DDDvet/PCU were chosen as these are the metrics defined by the EMA. The mg/PCU is currently used in the EMA’s European Surveillance Veterinary Antimicrobial Consumption reports [20] and by the electronic Medicines Database for pigs in the UK [44]. A major limitation of the mg/PCU is that it does not account for differences in potency between antimicrobials which is problematic if large number of farms are using highly potent drugs such as fluoroquinolones, cephalosporins or long-acting macrolides. Using a dose-based metric such as the DDDvet/PCU, likely to be used by the EMA once species-specific AMU data becomes available [17], can overcome this limitation. Nevertheless, the results for both indicators were remarkably similar which is in agreement with other studies in this field [45]. Detailed information on the length of production stages and accurate data on when AMs were administered were not available to this study, and therefore classical therapy incidence metrics (e.g. treatment incidence; [46]) could not be computed. Similarly, detailed data on the indications for use are not recorded by NAMUD. Such metrics would be very valuable from an epidemiological perspective, and are available to other, longer established monitoring programmes (SDa [35]). While this represents a potential point of improvement for the NAMUD project, the results presented demonstrate its importance and usefulness in monitoring and benchmarking AMU in Irish pig farming.

## Conclusions

This exploration of AMU in pigs in Ireland revealed significant reductions in overall use from 2019 to 2022, but signs of an increasing trend in 2023, which coincides with national-level One Health policy and international bans. The use of HPCIAs has also declined over the study period. Additionally, there was evidence of a significant decline in the use of oral premixes, coinciding with policy change. Herd production practices appear to be associated with AMU. These data are important for tracking the impact of national policies to reduce AMU in pig farming. Future longitudinal analysis of monitoring data will be needed to ensure that positive outcomes accrue and to understand whether/how these changes ultimately impact the emergence, dissemination and maintenance of AMR on Irish pig farms [47].

## Electronic supplementary material

Below is the link to the electronic supplementary material.

Supplementary Material 1

## Data Availability

The anonymized and aggregated data that supports the findings of this study are available on request from the first author (AMUPig@agriculture.gov.ie). The data are not publicly available due to privacy or ethical restrictions, and in keeping with agreements outlined in the National Antimicrobial Usage Database for Pigs (see: gov.ie– Pigs (www.gov.ie)) and Bord Bia Pigmeat Quality Assurance Scheme (PQAS Rev 4.1 Producer Summary Doc.pdf (bordbia.ie)).

## Abbreviations

AM: Antimicrobial
AMR: Antimicrobial resistance
AMU: Antimicrobial usage
GSEM: Generalised structural equation modelling
GEE: Generalised estimating equation
HPCIA: High priority critically important antimicrobial

## References

[1] Roca I, Akova M, Baquero F, Carlet J, Cavaleri M, Coenen S et al. The global threat of antimicrobial resistance: science for intervention. New microbes and new infections. 2015;6:22– 9. 10.1016/j.nmni.2015.02.007 PMC4446399 26029375

[2] O’Neill J. Tackling drug-resistant infections globally: final report and recommendations. 2016.

[3] Robinson TP, Bu D, Carrique-Mas J, Fèvre EM, Gilbert M, Grace D, et al. Antibiotic resistance is the quintessential one health issue. Trans R Soc Trop Med Hyg. 2016;110(7):377–80. 27475987 10.1093/trstmh/trw048 PMC4975175

[4] Van Boeckel TP, Brower C, Gilbert M, Grenfell BT, Levin SA, Robinson TP et al. Global trends in antimicrobial use in food animals. Proceedings of the National Academy of Sciences. 2015;112(18):5649-54. 10.1073/pnas.1503141112 PMC4426470 25792457

[5] Tiseo K, Huber L, Gilbert M, Robinson TP, Van Boeckel TP. Global trends in antimicrobial use in food animals from 2017 to 2030. Antibiotics. 2020;9(12):918. 33348801 10.3390/antibiotics9120918 PMC7766021

[6] Duarte ASR, Pessoa J, Attauabi M, Lindegaard M, Sönksen UW. DANMAP 2023: use of antimicrobial agents and occurrence of antimicrobial resistance in bacteria from food animals, food and humans in Denmark. 2024.

[7] ANSES. Sales of Veterinary Medicinal Products Containing Antimicrobials in France in 2022. Annual Report. ANSES-ANMV France. 2023.

[8] VMD. UK veterinary antibiotic resistance and sales surveillance report (UK-VARSS 2022). UK Government. 2023.

[9] SDa. Usage of antibiotics in agricultural livestock in the Netherlands in 2023. Utrecht, the Netherlands: Netherlands Veterinary Medicines Institute; 2024.

[10] Wissmann R, Kümmerlen D, Echtermann T. Trends in antimicrobial usage on Swiss pig farms from 2018 to 2021: based on an electronic treatment journal. Antibiotics. 2024;13(9):831. 39335005 10.3390/antibiotics13090831 PMC11440108

[11] Lopes Antunes AC, Jensen VF. Close to a decade of decrease in antimicrobial usage in Danish pig production–evaluating the effect of the yellow card scheme. Front Veterinary Sci. 2020;7:109. 10.3389/fvets.2020.00109 PMC7067903 32211427

[12] Van Boeckel TP, Glennon EE, Chen D, Gilbert M, Robinson TP, Grenfell BT, et al. Reducing antimicrobial use in food animals. Science. 2017;357(6358):1350–2. 28963240 10.1126/science.aao1495 PMC6510296

[13] EU. Regulation (EU) 2019/6 of the European Parliament and of the Council of 11 December 2018 on veterinary medicinal products and repealing directive 2001/82/EC. Official Journal of the European Union 2019.

[14] EU. Regulation (EU) 2019/4 of the European Parliament and of the Council. Official J Eur Union. 2019.

[15] More SJ, McCoy F, McAloon CI. The new veterinary medicines regulation: rising to the challenge. Ir Veterinary J. 2022;75(1):1–5. 10.1186/s13620-022-00209-6 PMC8812186 35115047

[16] DAFM. National Antimicrobial Usage Database for Pigs. https://www.govie/en/publication/fc9b3-pigs-farming-sectors/#national-antimicrobial-usage-database-for-pigs. 2019.

[17] DAFM. How to input antibiotic usage data on Www.agfood.ie for your pig herd. Department of Agriculture, Food and the Marine, Government of Ireland; 2021.

[18] Bolton J, O’Neill L, Garvan C, Byrne A. Herd-Level antimicrobial use in pig herds in Ireland (2019–2022): Temporal trends in consumption and composition. SSRN 2024(4763542).

[19] O’Neill L, Rodrigues da Costa M, Leonard FC, Gibbons J, Calderón Díaz JA, McCutcheon G, et al. Quantification, description and international comparison of antimicrobial use on Irish pig farms. Porcine Health Manage. 2020;6(1):1–14. 10.1186/s40813-020-00166-y PMC7549222 33062293

[20] EMA. Guidance on collection and provision of national data on antimicrobial use by animal species/categories. 2018.

[21] EMA. Report from the workshop on collection of data on antimicrobial agents by species and on unit(s) of measurement. Eur Med Agency. 2011(https://www.ema.europa.eu/en/documents/report/workshop-collection-data-antimicrobial-agents-species-and-units-measurement_en.pdf)

[22] EMA E. Defined daily doses for animals (DDDvet) and defined course doses for animals (DCDvet): European Surveillance of Veterinary Antimicrobial Consumption (ESVAC). European Surveillance of Veterinary Antimicrobial Consumition (ESVAC). 2016;44.

[23] StataCorp. Stata statistical software: release 16. MP - Parallel edition college station. TX: StataCorp LLC; 2019.

[24] Cui J. QIC program and model selection in GEE analyses. Stata J. 2007;7(2):209–20.

[25] Hardin JW, Hilbe JM. Generalized estimating equations. chapman and hall/CRC; 2002.

[26] Dohoo IR, Martin W, Stryhn HE. Veterinary epidemiologic research: UPEI, Canada; 2010.

[27] Chui KK, Webb P, Russell RM, Naumova EN. Geographic variations and Temporal trends of Salmonella-associated hospitalization in the US elderly, 1991–2004: a time series analysis of the impact of HACCP regulation. BMC Public Health. 2009;9(1):1–10. 19958556 10.1186/1471-2458-9-447 PMC2799411

[28] Afema JA, Davis MA, Sischo WM. Antimicrobial use policy change in pre-weaned dairy calves and its impact on antimicrobial resistance in commensal Escherichia coli: a cross sectional and ecological study. BMC Microbiol. 2019;19(1):1–14. 31514734 10.1186/s12866-019-1576-6 PMC6739941

[29] Xu J, Sangthong R, McNeil E, Tang R, Chongsuvivatwong V. Antibiotic use in chicken farms in Northwestern China. Antimicrob Resist Infect Control. 2020;9(1):1–9. 31921416 10.1186/s13756-019-0672-6 PMC6947973

[30] Pope R. The spotlight: Meet STATA’s new Xtmlogit command. Stata News. 2014;29(2):1–2.

[31] WHO. Critically important antimicrobials for human medicine. 2019.

[32] WHO. List of medically important antimicrobials: a risk management tool for mitigating antimicrobial resistance due to non-human use. Geneva: World Health Organization; 2024.

[33] CSO. Pig survey June 2022. Central Statistics Office, Ireland. 2022.

[34] Urban D, Chevance A, Fourès F. Suivi des ventes de médicaments vétérinaires contenant des antimicrobiens en France en 2022. Rapport annuel. 2023.

[35] SDa. Usage of Antibiotics in Agricultural Livestock in the Netherlands in 2022. https://www.autoriteitdiergeneesmiddelennl/english. 2023.

[36] O’Neill L, Calderón Díaz JA, Rodrigues da Costa M, Oakes S, Leonard FC, Manzanilla EG. Risk factors for antimicrobial use on Irish pig farms. Animals. 2021;11(10):2828. 34679849 10.3390/ani11102828 PMC8532697

[37] Postma M, Backhans A, Collineau L, Loesken S, Sjölund M, Belloc C, et al. Evaluation of the relationship between the biosecurity status, production parameters, herd characteristics and antimicrobial usage in farrow-to-finish pig production in four EU countries. Porcine Health Manage. 2016;2(1):1–11. 10.1186/s40813-016-0028-z PMC5382489 28405435

[38] iNAP. Policy on highest priority critically important antimicrobials 1st revision. Dublin: Department of Agriculture, Food and the Marine; 2020.

[39] Van der Fels-Klerx H, Puister-Jansen L, Van Asselt E, Burgers S. Farm factors associated with the use of antibiotics in pig production. J Anim Sci. 2011;89(6):1922–9. 21606448 10.2527/jas.2010-3046

[40] Hemme M, Ruddat I, Hartmann M, Werner N, van Rennings L, Käsbohrer A, et al. Antibiotic use on German pig farms-A longitudinal analysis for 2011, 2013 and 2014. PLoS ONE. 2018;13(7):e0199592. 29969477 10.1371/journal.pone.0199592 PMC6029768

[41] Sarrazin S, Joosten P, Van Gompel L, Luiken RE, Mevius DJ, Wagenaar JA, et al. Quantitative and qualitative analysis of antimicrobial usage patterns in 180 selected farrow-to-finish pig farms from nine European countries based on single batch and purchase data. J Antimicrob Chemother. 2019;74(3):807–16. 30544242 10.1093/jac/dky503

[42] Lekagul A, Tangcharoensathien V, Yeung S. Patterns of antibiotic use in global pig production: a systematic review. Veterinary Anim Sci. 2019;7:100058. 10.1016/j.vas.2019.100058 PMC7386699 32734079

[43] Moreno M. Survey of quantitative antimicrobial consumption in two different pig finishing systems. Vet Rec. 2012;171(13):325. 22915683 10.1136/vr.100818

[44] UK-VARSS. Veterinary antibiotic resistance and sales surveillance report (UK-VARSS 2023). New Haw, Addlestone: Veterinary Medicines Directorate; 2024.

[45] O’Neill L, Rodrigues da Costa M, Leonard F, Gibbons J, Calderon Diaz JA, McCutcheon G, et al. Does the use of different indicators to benchmark antimicrobial use affect farm ranking? Front Veterinary Sci. 2020;7:558793. 10.3389/fvets.2020.558793 PMC7590364 33195531

[46] Sjölund M, Postma M, Collineau L, Lösken S, Backhans A, Belloc C, et al. Quantitative and qualitative antimicrobial usage patterns in farrow-to-finish pig herds in Belgium, France, Germany and Sweden. Prev Vet Med. 2016;130:41–50. 27435645 10.1016/j.prevetmed.2016.06.003

[47] Byrne AW, Garvan C, Bolton J, Naranjo-Lucena A, Madigan G, McElroy M, et al. Antimicrobial resistance in Escherichia coli isolated from pigs and associations with aggregated antimicrobial usage in Ireland: A herd‐level exploration. Zoonoses Public Health. 2024;71(1):71–83. 37899534 10.1111/zph.13086

