# Supplementary material for: Antimicrobial use in pig herds in Ireland: analysis of a national database (2019–2023)
Source: Porcine Health Manag. 2025 May 2;11:24. doi: 10.1186/s40813-025-00438-5 (PMC12048958; doi:10.1186/s40813-025-00438-5)
Supplement: Supplementary file 1 — Supplementary Material 1 [file 40813_2025_438_MOESM1_ESM.docx]

**Supplementary material**

Section 1:

**Population Correction Unit**

The population correction unit (PCU) is the denominator used by the European Medicnes Agency (EMA) in its ESVAC reports on sales of veterinary antimicrobials in the EU and EEA ([EMA, 2011](#EMA2011)). The PCU assigns a standardised weight to each species and to sub-categories where applicable. In pigs, standard weights of 25 kg, 65 kg and 240 kg are assigned to weaner pigs, fattening (finisher) pigs and sows respectively ([EMA, 2011](#EMA2011)). It is specifically calculated using the numbers of breeding animals and the numbers of animals sent to slaughter or exported during the period at risk.

**DDDvet/PCU**

The differences in potency between the various antimicrobials were accounted for using the defined daily dose (DDDvet) for each active ingredient as defined by the European Medicines Agency (EMA, 2016). The amounts used of each antimicrobial were converted to ‘treatable kilograms’ (TK) based on the relevant DDDvet as outlined in the equation below. ‘Treatable kilograms’ represents the number of kilograms of pig which can be treated with the given amount of antimicrobial if the Defined Daily Dose is used. This calculation is based on the definition outlined by the Netherlands Veterinary Medicines Institute ([SDa, 2021](#SDa2021)):

$$treatable kilograms (TK\text{DDDvet})=\frac{amount of antimicrobial used \left( \mathrm{mg} \right)}{\mathrm{DDD}_{\mathrm{vet}} \left( mg/kg \right)}$$

Antimicrobials without an assigned DDD_vet_ (tulathromycin and tildipirosin) were assigned a DDDvet based on the defined DDDA and long-acting factors defined by Postma *et al.* (2015).

The DDDvet/PCU is calculated by dividing the TK$\text{DDDvet}$ by the PCU demonitor calculated at farm level.

**Figure S1:** Herd participation and population size over study period**.**

**Figure S2:** Trends in AMU (mg/kg PCU) across herds for each quarter for higher usage antimicrobials (solid line) over the study period (2019-2023) and the number of herds reporting usage of the AMD (circles). Note, high variability where contributing number of herds are low.

**Figure S3:** Trends in AMU (mg/kg PCU) across herds for each quarter for medium usage antimicrobials (solid line) over the study period (2019-2023) and the number of herds reporting usage of the AMD (circles). Note, high variability where contributing number of herds are low.

**Figure S4:** Trends in AMU (mg/kg PCU) across herds for each quarter for low usage antimicrobials (solid line) over the study period (2019-2023) and the number of herds reporting usage of the AMD (circles). Note, high variability where contributing number of herds are low.

**Figure S5:** Trends in AMU (mg/kg PCU) across herds for each quarter for very low usage antimicrobials (solid line) over the study period (2019-2023) and the number of herds reporting usage of the AMD (circles). Note, high variability where contributing number of herds are low.

**Fig. S6:** Predicted change in AMU over quarters for 57 herds that contributed AMU data for every quarter of the timeseries, controlling for herd type and size. Points with error bars: predictions from a categorical model with associated 95% confidence intervals.

**Fig. S7:** Predicted change in AMU over quarters modelled as three linear splines for 57 herds that contributed AMU data for every quarter of the timeseries, controlling for herd type and size. Points with error bars: predictions from a linear spline model with associated 95% confidence intervals.

**Fig. S8:** Predicted probability from a multinominal mixed effect model, fitted using the Generalised Structural Equation Modelling (SEM) framework with the predicted probability of usage for oral, parenteral and oral premix administration of AMs. Diamond= parenteral; square = premix; circle = oral.

**Table S1:** Summary table of AMU (mg/kg) across different production unit types and AM. Usage is measured as the total active substance per herd per quarter divided by the total weight per herd per quarter.

| Active Ingredient | Integrated unit | Breeder to Weaner unit | Finisher/fattening unit | Other | Total |
| --- | --- | --- | --- | --- | --- |
| AMOXICILLIN |  |  |  |  |  |
| Mean | 17.2 | 39.4 | 13.0 | 10.5 | 18.5 |
| Median | 2.2 | 3.7 | 0.7 | 1.4 | 1.9 |
| 25th%ile | 0.7 | 1.3 | 0.2 | 0.6 | 0.6 |
| 75th%ile | 17.1 | 29.7 | 4.6 | 2.3 | 14.9 |
| APRAMYCIN |  |  |  |  |  |
| Mean | 6.8 | 11.9 | 0.6 |  | 7.4 |
| Median | 2.0 | 4.0 | 0.5 |  | 2.1 |
| 25th%ile | 0.7 | 1.0 | 0.0 |  | 0.8 |
| 75th%ile | 6.0 | 14.8 | 1.2 |  | 6.7 |
| BENZYLPENICILLIN |  |  |  |  |  |
| Mean | 2.2 | 2.5 | 1.0 | 3.7 | 2.0 |
| Median | 1.1 | 1.2 | 0.5 | 1.5 | 1.0 |
| 25th%ile | 0.5 | 0.5 | 0.2 | 0.5 | 0.4 |
| 75th%ile | 2.4 | 3.7 | 1.3 | 8.5 | 2.2 |
| CEFQUINOME |  |  |  |  |  |
| Mean | 0.1 |  |  |  | 0.1 |
| Median | 0.1 |  |  |  | 0.1 |
| 25th%ile | 0.1 |  |  |  | 0.1 |
| 75th%ile | 0.2 |  |  |  | 0.2 |
| CEFTIOFUR |  |  |  |  |  |
| Mean | 0.2 | 0.1 | 0.2 |  | 0.2 |
| Median | 0.2 | 0.1 | 0.1 |  | 0.2 |
| 25th%ile | 0.1 | 0.0 | 0.0 |  | 0.1 |
| 75th%ile | 0.3 | 0.1 | 0.2 |  | 0.3 |
| CHLORTETRACYCLINE |  |  |  |  |  |
| Mean | 96.0 | 153.6 | 119.6 | 82.8 | 105.7 |
| Median | 49.6 | 89.8 | 50.2 | 55.8 | 52.2 |
| 25th%ile | 16.9 | 22.8 | 7.8 | 28.8 | 16.1 |
| 75th%ile | 123.1 | 183.6 | 184.4 | 134.5 | 140.8 |
| COLISTIN |  |  |  |  |  |
| Mean | 0.3 | 0.5 |  |  | 0.3 |
| Median | 0.4 | 0.5 |  |  | 0.4 |
| 25th%ile | 0.2 | 0.5 |  |  | 0.4 |
| 75th%ile | 0.4 | 0.5 |  |  | 0.4 |
| DIHYDROSTREPTOMYCIN |  |  |  |  |  |
| Mean | 1.5 | 2.4 | 0.9 | 0.5 | 1.5 |
| Median | 0.9 | 0.6 | 0.4 | 0.5 | 0.8 |
| 25th%ile | 0.4 | 0.3 | 0.1 | 0.1 | 0.3 |
| 75th%ile | 1.8 | 1.5 | 1.2 | 0.9 | 1.7 |
| DOXYCYCLINE |  |  |  |  |  |
| Mean |  | 6.5 | 58.6 |  | 48.2 |
| Median |  | 6.5 | 54.1 |  | 41.6 |
| 25th%ile |  | 6.5 | 27.6 |  | 13.6 |
| 75th%ile |  | 6.5 | 89.6 |  | 66.7 |
| ENROFLOXACIN |  |  |  |  |  |
| Mean | 0.3 | 0.4 | 0.2 | 0.1 | 0.3 |
| Median | 0.1 | 0.2 | 0.1 | 0.1 | 0.1 |
| 25th%ile | 0.0 | 0.1 | 0.0 | 0.1 | 0.0 |
| 75th%ile | 0.3 | 0.4 | 0.2 | 0.1 | 0.3 |
| FLORFENICOL |  |  |  |  |  |
| Mean | 13.1 | 2.9 | 3.6 | 20.6 | 11.3 |
| Median | 2.0 | 2.4 | 0.3 | 20.6 | 1.9 |
| 25th%ile | 0.7 | 1.2 | 0.1 | 20.6 | 0.5 |
| 75th%ile | 8.8 | 4.2 | 4.6 | 20.6 | 7.5 |
| GENTAMICIN |  |  |  |  |  |
| Mean | 0.0 |  |  |  | 0.0 |
| Median | 0.0 |  |  |  | 0.0 |
| 25th%ile | 0.0 |  |  |  | 0.0 |
| 75th%ile | 0.1 |  |  |  | 0.1 |
| LINCOMYCIN |  |  |  |  |  |
| Mean | 0.5 | 1.4 | 0.3 | 0.2 | 0.6 |
| Median | 0.2 | 0.6 | 0.2 | 0.1 | 0.2 |
| 25th%ile | 0.1 | 0.2 | 0.1 | 0.0 | 0.1 |
| 75th%ile | 0.5 | 1.6 | 0.3 | 0.3 | 0.5 |
| MARBOFLOXACIN |  |  |  |  |  |
| Mean | 0.2 | 0.3 | 1.9 | 0.4 | 0.5 |
| Median | 0.1 | 0.1 | 0.1 | 0.4 | 0.1 |
| 25th%ile | 0.0 | 0.0 | 0.0 | 0.3 | 0.0 |
| 75th%ile | 0.2 | 0.7 | 0.1 | 0.5 | 0.3 |
| NEOMYCIN |  |  |  |  |  |
| Mean | 11.7 | 7.7 | 34.3 |  | 13.0 |
| Median | 7.2 | 5.1 | 20.4 |  | 7.5 |
| 25th%ile | 2.3 | 2.1 | 13.2 |  | 2.4 |
| 75th%ile | 16.2 | 12.5 | 44.2 |  | 16.3 |
| OXYTETRACYCLINE |  |  |  |  |  |
| Mean | 1.1 | 1.5 | 3.2 | 1.0 | 1.5 |
| Median | 0.5 | 1.1 | 0.3 | 0.7 | 0.5 |
| 25th%ile | 0.2 | 0.7 | 0.2 | 0.3 | 0.2 |
| 75th%ile | 1.0 | 1.6 | 1.8 | 1.6 | 1.2 |
| PAROMOMYCIN |  |  |  |  |  |
| Mean | 1.1 | 2.0 | 7.0 |  | 1.6 |
| Median | 0.7 | 1.1 | 0.1 |  | 0.7 |
| 25th%ile | 0.3 | 0.4 | 0.0 |  | 0.3 |
| 75th%ile | 1.5 | 2.4 | 0.1 |  | 1.6 |
| SPECTINOMYCIN |  |  |  |  |  |
| Mean | 0.7 | 1.5 | 0.3 | 0.8 | 0.8 |
| Median | 0.3 | 0.5 | 0.2 | 0.7 | 0.3 |
| 25th%ile | 0.1 | 0.2 | 0.1 | 0.2 | 0.1 |
| 75th%ile | 0.6 | 1.4 | 0.3 | 1.3 | 0.7 |
| SULFADIAZINE |  |  |  |  |  |
| Mean | 44.9 | 44.4 | 59.9 | 10.0 | 45.8 |
| Median | 15.5 | 16.4 | 29.1 | 1.5 | 16.4 |
| 25th%ile | 3.2 | 1.2 | 12.6 | 0.9 | 3.0 |
| 75th%ile | 45.9 | 44.5 | 73.7 | 1.8 | 47.5 |
| SULFADOXINE |  |  |  |  |  |
| Mean | 0.5 |  |  |  | 0.5 |
| Median | 0.3 |  |  |  | 0.3 |
| 25th%ile | 0.2 |  |  |  | 0.2 |
| 75th%ile | 0.7 |  |  |  | 0.7 |
| TIAMULIN |  |  |  |  |  |
| Mean | 10.4 | 21.2 | 33.0 | 41.9 | 19.6 |
| Median | 2.3 | 1.4 | 1.1 | 29.3 | 1.5 |
| 25th%ile | 0.5 | 1.1 | 0.3 | 15.4 | 0.4 |
| 75th%ile | 11.1 | 7.3 | 9.1 | 47.3 | 12.0 |
| TILDIPIROSIN |  |  |  |  |  |
| Mean | 0.1 |  |  |  | 0.1 |
| Median | 0.1 |  |  |  | 0.1 |
| 25th%ile | 0.0 |  |  |  | 0.0 |
| 75th%ile | 0.1 |  |  |  | 0.1 |
| TILMICOSIN |  |  |  |  |  |
| Mean | 22.6 | 16.9 | 19.2 | 1.6 | 21.5 |
| Median | 11.5 | 6.2 | 6.3 | 1.6 | 10.8 |
| 25th%ile | 4.5 | 2.3 | 2.9 | 1.6 | 4.0 |
| 75th%ile | 23.6 | 24.4 | 35.5 | 1.6 | 24.0 |
| TRIMETHOPRIM |  |  |  |  |  |
| Mean | 8.7 | 8.9 | 12.0 | 2.0 | 8.9 |
| Median | 3.0 | 3.3 | 5.8 | 0.3 | 3.1 |
| 25th%ile | 0.5 | 0.2 | 2.5 | 0.2 | 0.5 |
| 75th%ile | 9.0 | 8.9 | 14.7 | 0.4 | 9.2 |
| TULATHROMYCIN |  |  |  |  |  |
| Mean | 0.2 | 0.3 | 0.1 | 0.0 | 0.2 |
| Median | 0.1 | 0.2 | 0.1 | 0.0 | 0.1 |
| 25th%ile | 0.0 | 0.0 | 0.0 | 0.0 | 0.0 |
| 75th%ile | 0.3 | 0.3 | 0.1 | 0.0 | 0.3 |
| TYLOSIN |  |  |  |  |  |
| Mean | 47.3 | 25.8 | 79.9 | 0.5 | 51.0 |
| Median | 6.6 | 11.2 | 40.9 | 0.5 | 9.8 |
| 25th%ile | 0.6 | 1.9 | 7.0 | 0.2 | 1.0 |
| 75th%ile | 30.3 | 35.9 | 89.7 | 0.8 | 42.1 |
| TYLVALOSIN |  |  |  |  |  |
| Mean | 7.8 |  |  |  | 7.8 |
| Median | 8.1 |  |  |  | 8.1 |
| 25th%ile | 4.3 |  |  |  | 4.3 |
| 75th%ile | 10.6 |  |  |  | 10.6 |

**Table S2:** Summary table of AMU (DDDvetPCU) across different production unit types and AM. Usage is measured as the total treated kilograms (DDDvetTK) per herd per quarter divided by the total weight per herd per quarter.

| Active Ingredient | Integrated unit | Breeder to Weaner unit | Finisher/fattening unit | Other | Total |
| --- | --- | --- | --- | --- | --- |
| AMOXICILLIN |  |  |  |  |  |
| Mean | 1.08 | 2.67 | 0.85 | 0.67 | 1.19 |
| Median | 0.22 | 0.37 | 0.08 | 0.15 | 0.19 |
| 25th%ile | 0.07 | 0.13 | 0.03 | 0.07 | 0.06 |
| 75th%ile | 1.11 | 2.08 | 0.44 | 0.20 | 1.03 |
| APRAMYCIN |  |  |  |  |  |
| Mean | 0.75 | 1.32 | 0.07 |  | 0.82 |
| Median | 0.23 | 0.44 | 0.06 |  | 0.24 |
| 25th%ile | 0.08 | 0.11 | 0.00 |  | 0.08 |
| 75th%ile | 0.66 | 1.64 | 0.13 |  | 0.74 |
| BENZYLPENICILLIN |  |  |  |  |  |
| Mean | 0.18 | 0.21 | 0.09 | 0.31 | 0.16 |
| Median | 0.09 | 0.10 | 0.04 | 0.12 | 0.08 |
| 25th%ile | 0.04 | 0.05 | 0.01 | 0.04 | 0.03 |
| 75th%ile | 0.20 | 0.31 | 0.11 | 0.71 | 0.18 |
| CEFQUINOME |  |  |  |  |  |
| Mean | 0.06 |  |  |  | 0.06 |
| Median | 0.05 |  |  |  | 0.05 |
| 25th%ile | 0.04 |  |  |  | 0.04 |
| 75th%ile | 0.08 |  |  |  | 0.08 |
| CEFTIOFUR |  |  |  |  |  |
| Mean | 0.27 | 0.10 | 0.07 |  | 0.25 |
| Median | 0.21 | 0.13 | 0.03 |  | 0.19 |
| 25th%ile | 0.10 | 0.01 | 0.01 |  | 0.07 |
| 75th%ile | 0.35 | 0.19 | 0.05 |  | 0.34 |
| CHLORTETRACYCLINE |  |  |  |  |  |
| Mean | 3.10 | 4.96 | 3.86 | 2.67 | 3.41 |
| Median | 1.60 | 2.90 | 1.62 | 1.80 | 1.68 |
| 25th%ile | 0.55 | 0.74 | 0.25 | 0.93 | 0.52 |
| 75th%ile | 3.97 | 5.92 | 5.95 | 4.34 | 4.54 |
| COLISTIN |  |  |  |  |  |
| Mean | 0.06 | 0.09 |  |  | 0.07 |
| Median | 0.08 | 0.09 |  |  | 0.08 |
| 25th%ile | 0.05 | 0.09 |  |  | 0.08 |
| 75th%ile | 0.08 | 0.09 |  |  | 0.08 |
| DIHYDROSTREPTOMYCIN |  |  |  |  |  |
| Mean | 0.08 | 0.12 | 0.05 | 0.02 | 0.07 |
| Median | 0.05 | 0.03 | 0.02 | 0.02 | 0.04 |
| 25th%ile | 0.02 | 0.02 | 0.01 | 0.00 | 0.02 |
| 75th%ile | 0.09 | 0.07 | 0.06 | 0.04 | 0.08 |
| DOXYCYCLINE |  |  |  |  |  |
| Mean |  | 0.59 | 5.33 |  | 4.38 |
| Median |  | 0.59 | 4.92 |  | 3.78 |
| 25th%ile |  | 0.59 | 2.51 |  | 1.24 |
| 75th%ile |  | 0.59 | 8.15 |  | 6.06 |
| ENROFLOXACIN |  |  |  |  |  |
| Mean | 0.09 | 0.12 | 0.06 | 0.03 | 0.09 |
| Median | 0.03 | 0.05 | 0.03 | 0.02 | 0.04 |
| 25th%ile | 0.01 | 0.02 | 0.01 | 0.02 | 0.01 |
| 75th%ile | 0.07 | 0.13 | 0.07 | 0.04 | 0.08 |
| FLORFENICOL |  |  |  |  |  |
| Mean | 1.35 | 0.31 | 0.36 | 2.06 | 1.16 |
| Median | 0.20 | 0.26 | 0.03 | 2.06 | 0.20 |
| 25th%ile | 0.07 | 0.13 | 0.01 | 2.06 | 0.05 |
| 75th%ile | 0.88 | 0.44 | 0.49 | 2.06 | 0.77 |
| GENTAMICIN |  |  |  |  |  |
| Mean | 0.03 |  |  |  | 0.03 |
| Median | 0.02 |  |  |  | 0.02 |
| 25th%ile | 0.01 |  |  |  | 0.01 |
| 75th%ile | 0.04 |  |  |  | 0.04 |
| LINCOMYCIN |  |  |  |  |  |
| Mean | 0.12 | 0.39 | 0.03 | 0.05 | 0.14 |
| Median | 0.02 | 0.10 | 0.02 | 0.01 | 0.02 |
| 25th%ile | 0.01 | 0.02 | 0.01 | 0.00 | 0.01 |
| 75th%ile | 0.06 | 0.52 | 0.03 | 0.04 | 0.07 |
| MARBOFLOXACIN |  |  |  |  |  |
| Mean | 0.10 | 0.17 | 0.96 | 0.21 | 0.23 |
| Median | 0.04 | 0.06 | 0.04 | 0.21 | 0.04 |
| 25th%ile | 0.02 | 0.02 | 0.01 | 0.15 | 0.02 |
| 75th%ile | 0.12 | 0.33 | 0.07 | 0.24 | 0.13 |
| NEOMYCIN |  |  |  |  |  |
| Mean | 0.48 | 0.36 | 1.38 |  | 0.54 |
| Median | 0.29 | 0.37 | 0.82 |  | 0.31 |
| 25th%ile | 0.11 | 0.11 | 0.53 |  | 0.12 |
| 75th%ile | 0.65 | 0.58 | 1.77 |  | 0.68 |
| OXYTETRACYCLINE |  |  |  |  |  |
| Mean | 0.14 | 0.19 | 0.43 | 0.13 | 0.20 |
| Median | 0.06 | 0.14 | 0.04 | 0.10 | 0.07 |
| 25th%ile | 0.03 | 0.09 | 0.02 | 0.04 | 0.03 |
| 75th%ile | 0.14 | 0.22 | 0.25 | 0.21 | 0.16 |
| PAROMOMYCIN |  |  |  |  |  |
| Mean | 0.04 | 0.08 | 0.27 |  | 0.07 |
| Median | 0.03 | 0.04 | 0.00 |  | 0.03 |
| 25th%ile | 0.01 | 0.02 | 0.00 |  | 0.01 |
| 75th%ile | 0.06 | 0.09 | 0.00 |  | 0.06 |
| SPECTINOMYCIN |  |  |  |  |  |
| Mean | 0.15 | 0.36 | 0.02 | 0.21 | 0.19 |
| Median | 0.03 | 0.10 | 0.02 | 0.17 | 0.04 |
| 25th%ile | 0.01 | 0.03 | 0.01 | 0.05 | 0.01 |
| 75th%ile | 0.08 | 0.26 | 0.02 | 0.32 | 0.10 |
| SULFADIAZINE |  |  |  |  |  |
| Mean | 1.96 | 1.94 | 2.61 | 0.46 | 2.00 |
| Median | 0.67 | 0.71 | 1.27 | 0.11 | 0.71 |
| 25th%ile | 0.18 | 0.08 | 0.55 | 0.04 | 0.17 |
| 75th%ile | 1.99 | 1.94 | 3.20 | 0.13 | 2.06 |
| SULFADOXINE |  |  |  |  |  |
| Mean | 0.04 |  |  |  | 0.04 |
| Median | 0.02 |  |  |  | 0.02 |
| 25th%ile | 0.02 |  |  |  | 0.02 |
| 75th%ile | 0.05 |  |  |  | 0.05 |
| TIAMULIN |  |  |  |  |  |
| Mean | 1.07 | 2.17 | 3.22 | 4.31 | 1.96 |
| Median | 0.24 | 0.11 | 0.09 | 3.02 | 0.14 |
| 25th%ile | 0.04 | 0.09 | 0.02 | 1.58 | 0.03 |
| 75th%ile | 1.15 | 0.76 | 0.94 | 4.87 | 1.22 |
| TILDIPIROSIN |  |  |  |  |  |
| Mean | 0.02 |  |  |  | 0.02 |
| Median | 0.02 |  |  |  | 0.02 |
| 25th%ile | 0.01 |  |  |  | 0.01 |
| 75th%ile | 0.03 |  |  |  | 0.03 |
| TILMICOSIN |  |  |  |  |  |
| Mean | 1.50 | 1.13 | 1.28 | 0.10 | 1.43 |
| Median | 0.77 | 0.42 | 0.42 | 0.10 | 0.72 |
| 25th%ile | 0.30 | 0.15 | 0.19 | 0.10 | 0.27 |
| 75th%ile | 1.58 | 1.63 | 2.37 | 0.10 | 1.60 |
| TRIMETHOPRIM |  |  |  |  |  |
| Mean | 1.86 | 1.90 | 2.55 | 0.45 | 1.91 |
| Median | 0.64 | 0.70 | 1.24 | 0.10 | 0.65 |
| 25th%ile | 0.12 | 0.08 | 0.53 | 0.04 | 0.12 |
| 75th%ile | 1.91 | 1.89 | 3.14 | 0.12 | 1.96 |
| TULATHROMYCIN |  |  |  |  |  |
| Mean | 0.09 | 0.10 | 0.03 | 0.01 | 0.09 |
| Median | 0.04 | 0.07 | 0.03 | 0.01 | 0.04 |
| 25th%ile | 0.01 | 0.01 | 0.00 | 0.01 | 0.01 |
| 75th%ile | 0.11 | 0.10 | 0.05 | 0.01 | 0.10 |
| TYLOSIN |  |  |  |  |  |
| Mean | 3.94 | 2.15 | 6.65 | 0.04 | 4.24 |
| Median | 0.55 | 0.94 | 3.41 | 0.04 | 0.78 |
| 25th%ile | 0.05 | 0.15 | 0.59 | 0.01 | 0.08 |
| 75th%ile | 2.53 | 2.99 | 7.48 | 0.06 | 3.51 |
| TYLVALOSIN |  |  |  |  |  |
| Mean | 2.17 |  |  |  | 2.17 |
| Median | 2.25 |  |  |  | 2.25 |
| 25th%ile | 1.20 |  |  |  | 1.20 |
| 75th%ile | 2.94 |  |  |  | 2.94 |

**Table S3:** Multivariable cluster-adjusted multinominal logit regression model of the administration of AMs in pig herds in Ireland over time (quarters 2019-2023)

| Administration route | exp(Beta) | p-value | lower 95%CI | upper 95%CI |
| --- | --- | --- | --- | --- |
|  |  |  |  |  |
| ORAL (EXCEPT PREMIX) | (base outcome) |  |  |  |
|  |  |  |  |  |
| PARENTERAL |  |  |  |  |
| year-quarter |  |  |  |  |
| 2019-1 | 0.932 | 0.406 | 0.789 | 1.100 |
| Referent | 1.000 |  |  |  |
| 2019-3 | 0.925 | 0.316 | 0.794 | 1.077 |
| 2019-4 | 0.934 | 0.426 | 0.791 | 1.104 |
| 2020-1 | 0.841 | 0.148 | 0.665 | 1.063 |
| 2020-2 | 0.804 | 0.081 | 0.630 | 1.027 |
| 2020-3 | 0.838 | 0.139 | 0.663 | 1.059 |
| 2020-4 | 0.834 | 0.134 | 0.658 | 1.057 |
| 2021-1 | 0.825 | 0.139 | 0.640 | 1.064 |
| 2021-2 | 0.870 | 0.295 | 0.671 | 1.129 |
| 2021-3 | 0.823 | 0.156 | 0.629 | 1.077 |
| 2021-4 | 0.826 | 0.164 | 0.630 | 1.082 |
| 2022-1 | 0.801 | 0.098 | 0.616 | 1.042 |
| 2022-2 | 0.841 | 0.223 | 0.637 | 1.111 |
| 2022-3 | 1.004 | 0.980 | 0.748 | 1.347 |
| 2022-4 | 0.945 | 0.667 | 0.730 | 1.224 |
| 2023-1 | 0.939 | 0.642 | 0.721 | 1.224 |
| 2023-2 | 0.942 | 0.671 | 0.715 | 1.241 |
| 2023-3 | 0.832 | 0.176 | 0.637 | 1.086 |
| 2023-4 | 0.886 | 0.338 | 0.691 | 1.136 |
| Herd type |  |  |  |  |
| Integrated unit (ref) | 1.000 |  |  |  |
| Breeder to Weaner unit | 0.615 | 0.010 | 0.424 | 0.891 |
| Finisher/fattening unit | 1.926 | 0.000 | 1.343 | 2.760 |
| Other | 1.877 | 0.139 | 0.815 | 4.322 |
| Herd size quartiles (size range) |  |  |  |  |
| 1 (<1442; Ref.) | 1.000 |  |  |  |
| 2 (1442-3157) | 0.652 | 0.007 | 0.476 | 0.892 |
| 3 (3158-6006) | 0.643 | 0.022 | 0.440 | 0.939 |
| 4 (6001-23056) | 0.647 | 0.015 | 0.456 | 0.919 |
|  |  |  |  |  |
| Constant | 8.066 | 0.000 | 5.477 | 11.878 |
|  |  |  |  |  |
| ORAL PREMIX |  |  |  |  |
| year-quarter |  |  |  |  |
| 2019-1 | 0.876 | 0.167 | 0.727 | 1.057 |
| Referent | 1.000 |  |  |  |
| 2019-3 | 0.896 | 0.233 | 0.749 | 1.073 |
| 2019-4 | 0.867 | 0.224 | 0.690 | 1.091 |
| 2020-1 | 0.802 | 0.130 | 0.602 | 1.067 |
| 2020-2 | 0.834 | 0.220 | 0.624 | 1.115 |
| 2020-3 | 0.843 | 0.248 | 0.630 | 1.127 |
| 2020-4 | 0.798 | 0.108 | 0.605 | 1.051 |
| 2021-1 | 0.788 | 0.122 | 0.583 | 1.065 |
| 2021-2 | 0.750 | 0.078 | 0.545 | 1.032 |
| 2021-3 | 0.702 | 0.028 | 0.513 | 0.962 |
| 2021-4 | 0.643 | 0.007 | 0.468 | 0.884 |
| 2022-1 | 0.602 | 0.002 | 0.437 | 0.829 |
| 2022-2 | 0.628 | 0.007 | 0.448 | 0.881 |
| 2022-3 | 0.640 | 0.011 | 0.453 | 0.905 |
| 2022-4 | 0.576 | 0.001 | 0.417 | 0.796 |
| 2023-1 | 0.666 | 0.015 | 0.479 | 0.925 |
| 2023-2 | 0.696 | 0.027 | 0.505 | 0.960 |
| 2023-3 | 0.571 | 0.001 | 0.414 | 0.788 |
| 2023-4 | 0.582 | 0.001 | 0.423 | 0.801 |
| Herd type |  |  |  |  |
| Integrated unit (ref) | 1.000 |  |  |  |
| Breeder to Weaner unit | 1.010 | 0.966 | 0.632 | 1.614 |
| Finisher/fattening unit | 1.423 | 0.138 | 0.893 | 2.270 |
| other | 2.712 | 0.109 | 0.801 | 9.180 |
| Herd size quartiles (size range) |  |  |  |  |
| 1 (<1442; Ref.) | 1.000 |  |  |  |
| 2 (1442-3157) | 0.829 | 0.418 | 0.527 | 1.304 |
| 3 (3158-6006) | 0.921 | 0.751 | 0.554 | 1.531 |
| 4 (6001-23056) | 0.948 | 0.832 | 0.577 | 1.556 |
|  |  |  |  |  |
| Constant | 1.999 | 0.009 | 1.192 | 3.351 |

**Table S4:** Multivariable multinomial logit regression model of the administration of AMs in pig herds in Ireland over time (quarters 2019-2023) with a random herd effect fitted using GSEM

| Administration route | exp(Beta) | p-value | lower 95%CI | upper 95%CI |
| --- | --- | --- | --- | --- |
|  |  |  |  |  |
| ORAL (EXCEPT PREMIX) | (base outcome) |  |  |  |
|  |  |  |  |  |
| PARENTERAL |  |  |  |  |
| year-quarter |  |  |  |  |
| 2019-1 | 0.961 | 0.831 | 0.665 | 1.388 |
| Referent | 1.000 |  |  |  |
| 2019-3 | 0.947 | 0.772 | 0.656 | 1.367 |
| 2019-4 | 1.022 | 0.906 | 0.717 | 1.457 |
| 2020-1 | 0.947 | 0.736 | 0.692 | 1.297 |
| 2020-2 | 0.899 | 0.505 | 0.656 | 1.230 |
| 2020-3 | 0.942 | 0.710 | 0.687 | 1.292 |
| 2020-4 | 0.926 | 0.635 | 0.676 | 1.270 |
| 2021-1 | 0.950 | 0.747 | 0.694 | 1.300 |
| 2021-2 | 0.962 | 0.811 | 0.703 | 1.318 |
| 2021-3 | 0.969 | 0.844 | 0.708 | 1.326 |
| 2021-4 | 1.013 | 0.936 | 0.741 | 1.385 |
| 2022-1 | 0.985 | 0.925 | 0.721 | 1.345 |
| 2022-2 | 0.987 | 0.937 | 0.718 | 1.357 |
| 2022-3 | 1.185 | 0.309 | 0.854 | 1.645 |
| 2022-4 | 1.143 | 0.421 | 0.825 | 1.583 |
| 2023-1 | 1.150 | 0.397 | 0.832 | 1.590 |
| 2023-2 | 1.134 | 0.450 | 0.819 | 1.570 |
| 2023-3 | 1.012 | 0.942 | 0.733 | 1.397 |
| 2023-4 | 1.129 | 0.470 | 0.813 | 1.568 |
| Herd type |  |  |  |  |
| Integrated unit (ref) | 1.000 |  |  |  |
| Breeder to Weaner unit | 0.625 | 0.087 | 0.365 | 1.071 |
| Finisher/fattening unit | 2.631 | 0.000 | 1.776 | 3.899 |
| Other | 2.192 | 0.206 | 0.649 | 7.401 |
| Herd size quartiles (size range) |  |  |  |  |
| 1 (<1442; Ref.) | 1.000 |  |  |  |
| 2 (1442-3157) | 0.663 | 0.001 | 0.517 | 0.851 |
| 3 (3158-6006) | 0.601 | 0.000 | 0.454 | 0.795 |
| 4 (6001-23056) | 0.598 | 0.002 | 0.434 | 0.823 |
|  |  |  |  |  |
| Constant | 10.892 | 0.000 | 7.349 | 16.144 |
|  |  |  |  |  |
| ORAL PREMIX |  |  |  |  |
| year-quarter |  |  |  |  |
| 2019-1 | 0.798 | 0.300 | 1.920 | 1.223 |
| Referent | 1.000 |  |  |  |
| 2019-3 | 0.888 | 0.300 | 1.721 | 1.357 |
| 2019-4 | 0.932 | 0.584 | 1.618 | 1.405 |
| 2020-1 | 0.823 | 0.736 | 1.750 | 1.184 |
| 2020-2 | 0.833 | 0.293 | 1.727 | 1.198 |
| 2020-3 | 0.860 | 0.325 | 1.677 | 1.239 |
| 2020-4 | 0.804 | 0.417 | 1.795 | 1.159 |
| 2021-1 | 0.809 | 0.242 | 1.779 | 1.165 |
| 2021-2 | 0.789 | 0.255 | 0.547 | 1.138 |
| 2021-3 | 0.747 | 0.205 | 1.930 | 1.078 |
| 2021-4 | 0.669 | 0.119 | 2.158 | 0.965 |
| 2022-1 | 0.602 | 0.032 | 2.397 | 0.869 |
| 2022-2 | 0.584 | 0.007 | 2.493 | 0.849 |
| 2022-3 | 0.626 | 0.005 | 2.355 | 0.923 |
| 2022-4 | 0.604 | 0.018 | 2.439 | 0.890 |
| 2023-1 | 0.683 | 0.011 | 2.144 | 0.999 |
| 2023-2 | 0.673 | 0.049 | 2.179 | 0.987 |
| 2023-3 | 0.585 | 0.043 | 2.507 | 0.857 |
| 2023-4 | 0.619 | 0.006 | 2.383 | 0.914 |
| Herd type |  |  |  |  |
| Integrated unit (ref) | 1.000 |  |  |  |
| Breeder to Weaner unit | 1.282 | 0.564 | 0.552 | 2.978 |
| Finisher/fattening unit | 1.275 | 0.428 | 0.699 | 2.325 |
| other | 5.176 | 0.066 | 0.898 | 29.838 |
| Herd size quartiles (size range) |  |  |  |  |
| 1 (<1442; Ref.) | 1.000 |  |  |  |
| 2 (1442-3157) | 1.096 | 0.579 | 0.794 | 1.513 |
| 3 (3158-6006) | 1.006 | 0.975 | 0.698 | 1.450 |
| 4 (6001-23056) | 1.042 | 0.848 | 0.684 | 1.588 |
|  |  |  |  |  |
| Constant | 0.993 | 0.979 | 0.587 | 1.680 |
